# Experimental test of fine-grained entropic uncertainty relation in the presence of quantum memory

**DOI:** 10.1038/s41598-019-45205-z

**Published:** 2019-06-19

**Authors:** Wei-Min Lv, Chao Zhang, Xiao-Min Hu, Yun-Feng Huang, Huan Cao, Jian Wang, Zhi-Bo Hou, Bi-Heng Liu, Chuan-Feng Li, Guang-Can Guo

**Affiliations:** 1 CAS Key Laboratory of Quantum Information, University of Science and Technology of China, Hefei, 230026 People’s Republic of China; 2 CAS Center For Excellence in Quantum Information and Quantum Physics, University of Science and Technology of China, Hefei, 230026 People’s Republic of China

**Keywords:** Optical physics, Quantum mechanics

## Abstract

The uncertainty principle, which gives the constraints on obtaining precise outcomes for incompatible measurements, provides a new vision of the real world that we are not able to realize from the classical knowledge. In recent years, numerous theoretical and experimental developments about the new forms of the uncertainty principle have been achieved. Among these efforts, one attractive goal is to find tighter bounds of the uncertainty relation. Here, using an all optical setup, we experimentally investigate a most recently proposed form of uncertainty principle—the fine-grained uncertainty relation assisted by a quantum memory. The experimental results on the case of two-qubit state with maximally mixed marginal demonstrate that the fine-graining method can help to get a tighter bound of the uncertainty relation. Our results might contribute to further understanding and utilizing of the uncertainty principle.

## Introduction

Since the original idea of uncertainty principle proposed by Heisenberg in 1927^1^, the uncertainty principle, one of the most remarkable features of quantum mechanics distinguishing from the classical physical world, has been studied for over ninety years with various forms of uncertainty relations achieved, such as standard deviation and entropy^2–4^. In recent years, there has been a great interest in the entropic uncertainty relations motivated by its important applications, such as quantum cryptography^5–8^, entanglement witnesses^9–12^ and quantum metrology^13^.

Soon after the first entropic uncertainty relation for pairs of non-degenerate observables given by Deutsch^14^, the popular version of uncertainty relation was conjectured and proved^15,16^, which can be extended to a pair of POVM measurements^17^

1 $$H(R)+H(S)\ge {\mathrm{log}}_{2}\frac{1}{c},$$

where *H*(*X*) denotes the Shannon entropy of the outcome probability distribution when *X* is measured. And *c* = *max*_*i*,*j*_|〈*a*_*i*_|*b*_*j*_〉|^2^, where |*a*_*i*_〉(|*b*_*j*_〉) represents the eigenvectors of the observables *R*(*S*), quantifying the complementary of the observables. Nevertheless, the lower bound of the inequality (1) can be improved in the presence of quantum memory^6^, which have been proved experimentally^10,11^. The entropic uncertainty relation with quantum memory is

2 $$S(R|B)+S(S|B)\ge {\mathrm{log}}_{2}\frac{1}{c}+S(A|B),$$

where the conditional von Neumann entropy *S*(*R*|*B*) of the state given by $\sum _{i}\,(|{\psi }_{i}\rangle \langle {\psi }_{i}|\otimes I){\rho }_{AB}(|{\psi }_{i}\rangle \langle {\psi }_{i}|\otimes I)$, with |*ψ*_*i*_〉 being the eigenstate of observable *R*, quantifies the uncertainty of the measurement outcomes of R with the information stored in a quantum memory *B*. The conditional von Neumann entropy *S*(*A*|*B*) quantifies the degree of correlation between the particle *A* and the quantum memory *B*. With the help of the Fano’s inequality^18^, the entropic uncertainty can be given by^11^

3 $$H({p}_{d}^{R})+H({p}_{d}^{S})\ge {\mathrm{log}}_{2}\frac{1}{c}+S(A|B),$$

where $H({p}_{d}^{R(S)})=-\,{p}_{d}^{R(S)}\,{\mathrm{log}}_{2}{p}_{d}^{R(S)}-(1-{p}_{d}^{R(S)}){\mathrm{log}}_{2}(1-{p}_{d}^{R(S)})$ is a binary entropy and ${p}_{d}^{R}({p}_{d}^{S})$ is the probability that Alice and Bob choose the same measurement of observables *R*(*S*) and get different outcomes.

Even though the entropic function is fairly appropriate in many cases, it is a rather coarse way to describe quantum uncertainty. Each entropy is just a function of the probability distribution of measurement outcomes as a whole, and it can not distinguish the uncertainty inherent in obtaining any combination of outcomes for different measurements. Considering these reasons, Oppenheim and Wehner proposed the so-called fine-grained uncertainty relation^19^. For a set of measurements *T* = {*t*} and the corresponding probability distribution *D* = {*p*(*t*)} over the measurements *T*, a series of inequalities with one for each combination of possible outcomes can be obtained by

4 $$\{P(\sigma ;\,{\bf{x}})\,:=\sum _{t=1}^{n}\,p(t)p{({x}^{(t)}|t)}_{\sigma }\le {\zeta }_{{\bf{x}}}|\forall \,{\bf{x}}\in {{\bf{B}}}^{\times n}\},$$

where $P(\sigma ;\,{\bf{x}})$ is the probability of one possible combination of measurement outcomes, written as a string ${\bf{x}}=({x}^{(1)},{x}^{(2)},\ldots ,{x}^{(n)})\in {{\bf{B}}}^{\times n}$ with $n=|T|$ representing the number of measurements, $p{({x}^{(t)}|t)}_{\sigma }$ is the probability of obtaining outcome ${x}^{(t)}$ when the measurement labelled *t* is performed on the system state $\sigma$, and ${\zeta }_{{\bf{x}}}=\mathop{{\rm{\max }}\,}\limits_{\sigma }\sum _{t=1}^{n}\,p(t)p{({x}^{(t)}|t)}_{\sigma }$, with the maximization taken over all states allowed on a particular system. Therefore, the uncertainty of measurement outcome occurs whenever ${\zeta }_{{\bf{x}}}\le 1$. Then the idea of fine-grained uncertainty relation was applied to the case of bipartite systems and established the interesting connection with nonlocality by Oppenheim and Wehner^19^. They considered the nonlocal retrieval game played by Alice and Bob, sharing a bipartite state, receiving a binary question from Chalice whose questions that sent to Alice and Bob is chosen completely randomly, and then giving their binary answers after measurements performed on their own qubit, respectively. Based on the winning condition which can be seen as a particular choice of measurement outcomes in the game, there is a maximum winning probability maximizing over the set of measurement settings chosen by Alice and Bob. There is a close connection between the game and uncertainty relation, in particular, every game can be considered as giving rise to an uncertainty relation, and vice versa.

It has been shown that the fine-grained uncertainty relation can be used to many physical issues of interest, for example, involving nonlocality to discriminate among classical, quantum, and superquantum correlations^19–21^, investigating steerability in theory and experiment with the ability to detect steering in all steerable two-qubit Werner states using only two measurement settings^22–24^, exploring the second law of thermodynamics and concluding that a violation of the uncertainty relation implies a violation of the thermodynamical law^25,26^, for the uncertainty relation in a relativistic regime suggesting that the uncertainty bound is dependent on the acceleration parameter and the choice of Unruh modes^27^. Besides, some other studies can be found in^28,29^.

Pramanik *et al*. apply the fine-graining strategy into the scenario with the presence of quantum memory and derive a new form of the uncertainty relation, in which the lower bound of entropic uncertainty corresponding to the measurement of two observables is determined by fine graining the possible measurement outcomes^30^. They consider a quantum game that Alice prepares a two-qubit state *ρ*_*AB*_, and sends one of the qubits to Bob as the quantum memory. Then Alice performs one of measurements, corresponding to the two observables *R* and *S* on her qubit, and informs Bob of the choice of the measurement. What Bob’s task is to minimize the uncertainty of the measurement outcomes, $H({p}_{d}^{R})+H({p}_{d}^{S})$. Their fine-grained uncertainty relation reads

5 $$H({p}_{d}^{R})+H({p}_{d}^{S})\ge H({p}_{d}^{{\sigma }_{Z}})+H({p}_{inf}^{S}).$$

The first term at the right hand side of inequality (5) is the entropy as they choose the measurement *R* = *σ*_*Z*_. And ${p}_{inf}^{S}$ is the infimum of ${P}_{d}^{S}$ as *S* ≠ *σ*_*Z*_, which is given by

6 $${p}_{inf}^{S}=\mathop{inf}\limits_{s\ne {\sigma }_{z}}\,\sum _{a,b}V(a,\,b)Tr[({A}_{S}^{a}\otimes {B}_{S}^{b}){\rho }_{AB}],$$

${A}_{S}^{a}({B}_{S}^{b})\,$is the projector of observable *S* with outcome *a*(*b*), and the condition function reads

7 $$\begin{array}{llll}V(a,b) & = & 1 & a\oplus b=1\\  & = & 0 & otherwise,\end{array}$$

which is the essence of the fine-grained strategy. The uncertainty relation (5) is independent of the choice of measurement settings because it optimizes the reduction of uncertainty quantified by the conditional Shannon entropy over all possible observables.

## Experimental Part

In this paper, we report an all-optical experiment to investigate the fine-grained uncertainty relation with the presence of a quantum memory, in the case of a two-qubit state with maximally mixed marginal, one qubit for the system under consideration, the other for the memory. Our results show that fine-graining actually gives a finer lower bound than previous course-graining entropic functions, that is, there is a gap between the attainable minimal uncertainty after fine-graining and the right hand side of inequality (3). We further make numerical simulations for the cases of different states, which also give similar conclusions.

**Figure 1:**
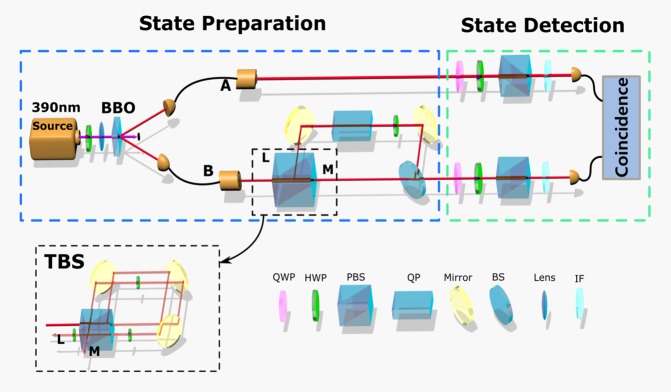
Experimental setup. There are two parts, state preparation and detection. For state preparation part: The Source contains the process that an ultrafast pulse, emitted from a mode-locked Ti:sapphire laser with 140 fs duration, 76 MHz repetition rate, and 780 nm central wavelength, passes through a frequency doubler to generate the 390 nm pulse. Then the ultraviolet pulse passes a sandwich-like BBO crystal to generate a pair of polarization-entangled photons, *A* and *B*, via spontaneous parametric down-conversion (SPDC) process. Photon A is directly measured by the detection part, while photon B passes through an unbalanced Mach-Zehnder interference (UMZ) set-up with one arm M introducing decoherence by adding a quartz plate (QP) and the path difference between the short and long arms of UMZ is about 0.15 m, corresponding to the time difference about 0.5 ns, which is larger than the coherence length of the photons and smaller than the coincidence window. The ratio of the relative amplitude of two arms L and M in the UMZ can be adjusted by a special designed tunable beam splitter (TBS) (black dotted line rectangle), which contains a polarizing beam splitter (PBS), three mirrors and three half-wave plates (HWPs). Taking a photon with the state *α*|*H*〉 + *β*|*V*〉 as an example. The photon is split into two paths, transmission (path 1) and reflection (path 2) in the loop of TBS when it arrives at the PBS, and the state of photon becomes *α*|*H*〉_1_ + *β*|*V*〉_2_, then they are coincident on the PBS after being transformed by the two HWPs set at the same angle *θ*_1_ = *θ*_2_ = *θ* respectively, as *α*(cos2*θ*|*H*〉_1_ + sin2*θ*|*V*〉_1_) + *β*(sin2*θ*|*H*〉_2_ − cos2*θ*|*V*〉_2_). So the states at the two output ports M and L of the TBS are cos2*θ*(*α*|*H*〉_1_ − *β*|*V*〉_2_) and sin2*θ*(*α*|*V*〉_1_ + *β*|*H*〉_2_), respectively. At last, after tilting the QWP at the M port (not shown in Fig. 1) and inserting a 45° HWP at the L port, the output amplitude ratio between M and L port of the TBS can be obtained, *M*:*L* = 1:tan2*θ*. For detection part, photon *A* and *B* pass through interference filters (IFs) with 8 nm and 3 nm respectively, before projective detections of photons are chosen by the angles of quarter-wave plates (QWPs) and HWPs. Then the coincidence detection with 6-ns coincidence window is performed in the coincidence detection unit.

The experiment setup is shown in Fig. 1. It can be divided into two parts, state preparation and detection. First we start with the state preparation. For experimental simplicity, we just consider the two-qubit case. Here, we choose to prepare a maximally mixed marginal state, given by $\rho =\frac{1}{4}\times ({I}_{4\times 4}+{\sum }_{j=1}^{3}{c}_{j}{\sigma }_{j}\otimes {\sigma }_{j})$, with *c*_*j*_ are real constants satisfying the constraint condition that the state *ρ* is physical. In the experiment, we use EPR source to generate a pair of polarization-entangled photons A and B, with maximally entangled state $\frac{1}{\sqrt{2}}(|HH\rangle +|VV\rangle )$. The photon *A* is directly transformed to the state detection part by a fiber, while the photon *B* passes through a TBS^31^. In order to prepare the target state which is determined by the parameters *c*_1_ = 0.6, *c*_2_ = −0.16, *c*_3_ = −0.24, we set the angles of the two HWPs in the loop of TBS, *θ*_1_ = *θ*_2_ ≈ 26°, which means about 62.1% of the entangled photon $\frac{1}{\sqrt{2}}(|HH\rangle +|VV\rangle )$ is transformed to $\frac{1}{\sqrt{2}}(|HV\rangle +|VH\rangle )$. Then the photon with the state $\frac{1}{\sqrt{2}}(|HV\rangle +|VH\rangle )$ is partly decohered into 0.177|*HV* + *VH*〉〈*HV* + *VH*| + 0.323(|*HV*〉〈*HV*| + |*VH*〉〈*VH*|) by inserting a 160*λ* (780 *nm*) quartz plate (QP) in the long arm before implementing incoherent superposition with the photon in the short arm. After state preparation, photon *A* and *B* pass through the 8-nm and 3-nm interference filters^32^, and the projective measurements ${A}_{S}^{a}=\frac{I+{(-1)}^{a}\hat{n}\cdot \overrightarrow{\sigma }}{2}$ and ${A}_{S}^{b}=\frac{I+{(-1)}^{b}\hat{n}\cdot \overrightarrow{\sigma }}{2}$ of the observable *S* are performed on the two photons *A* and *B* respectively, where the observable *S* is parameterized by $S=\hat{n}\cdot \overrightarrow{\sigma }$, $\hat{n}=\{sin\theta cos\varphi ,sin\theta sin\varphi ,cos\theta \}$, $\overrightarrow{\sigma }=\{{\sigma }_{x},{\sigma }_{y},{\sigma }_{z}s\}$ are the Pauli matrices, and *a* (*b*) takes the value either 0 or 1. So we can get the probability ${P}_{d}^{S}$ that satisfying the winning condition *a* ⊕ *b* = 1, which means the uncertainty $H({P}_{d}^{S})$. By changing the angles of QWPs and HWPs in the state detection part, the projection direction $\hat{n}$ can be taken all over. Besides, we also need to perform state tomography measurements to evaluate the Berta’s uncertainty bound. And these measurement results help to verify the gap between the fine-grained bound and Berta’s bound.

## Results

**Figure 2:**
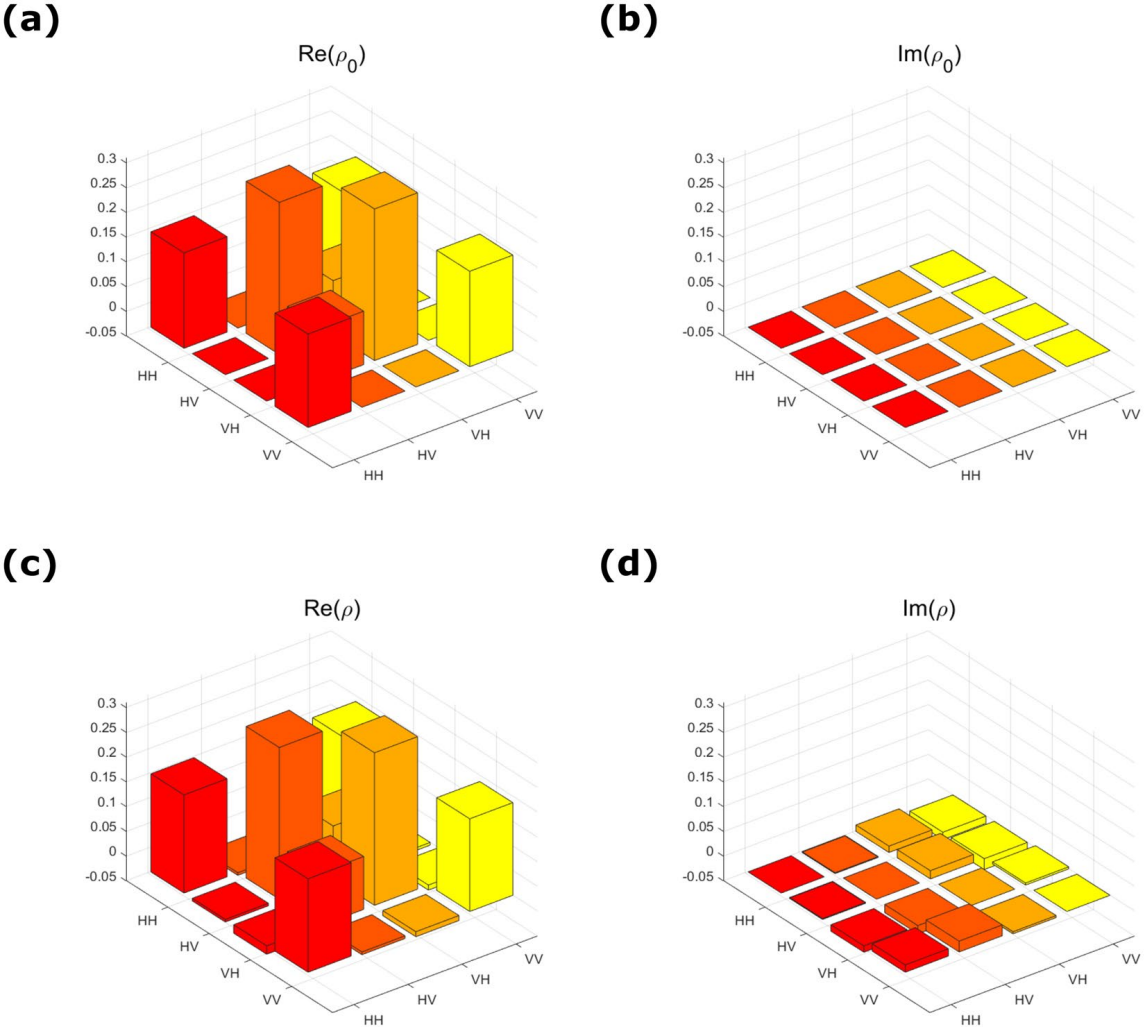
The density matrix of target state *ρ*_0_ and experimental state *ρ*. (**a**) and (**b**) show the real and imaginary part of target state *ρ*_0_, *Re*(*ρ*_0_) and *Im*(*ρ*_0_) respectively. (**c**) and (**d**) show the real and imaginary part of prepared state *ρ* in our experiment, *Re*(*ρ*) and *Im*(*ρ*) respectively.

Figure 2 shows the density matrix of the prepared two-qubit state *ρ*, and the target state. The density matrix of the prepared state is reconstructed by quantum state tomography^33^, which shows a rather high fidelity with the fidelity $F=Tr[\sqrt{\sqrt{\rho }{\rho }_{0}\sqrt{\rho }}]\approx 0.9967\pm 0.0013$.

**Figure 3:**
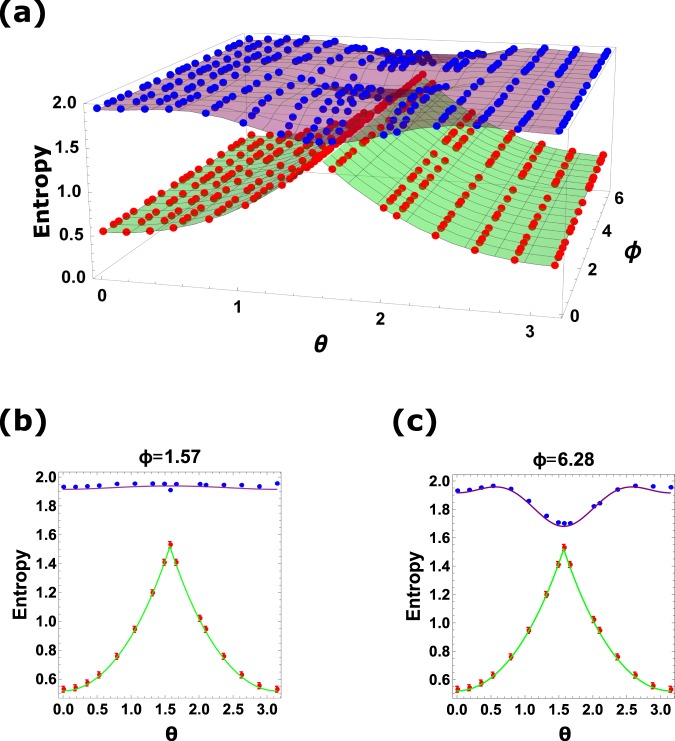
Experimental results and theoretical predictions. (**a**) Theoretical predictions and experimental results for the Berta’s uncertainty bound and fine-grained uncertainty relation. The purple and green curve surfaces show the theoretical predictions of fine-grained uncertainty $H({p}_{d}^{{\sigma }_{Z}})+H({p}_{d}^{S})$ and Berta’s uncertainty bound ${\mathrm{log}}_{2}\frac{1}{c}+S(A|B)$ for different *θ* and *ϕ* respectively (*θ* and *ϕ* are in the unit of radian). The blue and red spheres represent the experimental results of them separately. (**b**) and (**c**) represent the special case when *ϕ* = 1.57 and *ϕ* = 6.28. The purple and green solid line represent the theoretical predictions with ideal state. The blue and red solid circles represent the corresponding experiment results. In this experiment, the minimum value of $H({p}_{d}^{S})$ occurs in this case for *θ* = 1.57, *ϕ* = 6.28, yielding the lower bound of inequality (5), $H({p}_{d}^{{\sigma }_{Z}})+H({p}_{inf}^{S})\approx 1.69$, and our experimental result is 1.70. It can be seen that our experimental results coincide with the theoretical predictions very well.

The experimental results and theoretical predictions of the Berta’s uncertainty bound^6^ and the fine-grained uncertainty relation are shown in Fig. 3. The purple and green curve surfaces indicate the theoretical predictions of fine-grained uncertainty and Berta’s uncertainty bound respectively. The discrete blue spheres represent the experimental results for fine-grained entropic uncertainty $H({p}_{d}^{{\sigma }_{Z}})+H({p}_{d}^{S})$. And the red spheres represent Berta’s uncertainty bound ${\mathrm{log}}_{2}\frac{1}{c}+S(A|B)$, which we can calculate with the reconstructed density matrix. We also choose two special cases where *ϕ* = 1.57 and *ϕ* = 6.28 respectively to observe the difference between the Berta’s uncertainty bound and the fine-grained uncertainty in detail. The minimum value of $H({p}_{d}^{S})$ occurs in this case for *θ* = 1.57, *ϕ* = 6.28, yielding the lower bound of $H({p}_{d}^{{\sigma }_{Z}})+H({p}_{inf}^{S})\approx 1.69$, and our experimental result is 1.70 ± 0.003, which clearly exhibits a gap above the corresponding Berta’s uncertainty bound 1.534 ± 0.019 calculated from the experimentally reconstructed density matrix. However, to experimentally verify the theoretical prediction, we need to consider the finite sample effect when experimentally evaluating the bounds, which leads to finite error bars in Fig. 3. The error bars are estimated from the standard deviations of the values calculated via the Monte Carlo method^34^. To obtain the error bars for evaluating a result from a set of measured coincidence counts {*N*_*i*_}, we repeatedly generate a set of random numbers on computer, which obey the Poisson distributions centered at the corresponding measured coincidence counts, and the widths of the Poisson distributions are set as mean of the counts {$\sqrt{{N}_{i}}$}. With each set of random numbers, we can evaluate the desired result one time. Then the error bars can be obtained by calculating the standard deviations of the results after repeating the above process for many times. The coincidence counting rate without polarization projection measurement in our experiment is about 5000 per second, and the data collection time for each measurement is 15 s. The repeating times in the Monte Carlo method is set to be 100. Finally, we get the error bars about ±0.003 for fine-grained entropic uncertainty and ±0.019 for the Berta’s uncertainty bound. These error bars are much smaller than the smallest gap 0.17 between the two kinds of uncertainty bounds, which demonstrate that finite sample effect here would not ruin the experimental results. To note that for every R and S, Berta’s uncertainty bound is also related to the density matrix of state, which contains more parameters than the fine-grained entropic uncertainty. This is the reason why the error bars of the Berta’s uncertainty bound are much larger than the fine-graining bound. From Fig. 3 we see that our experimental results agree with the theoretical predictions very well, which proves the fine-grained uncertainty relation (5) and shows that the Berta’s uncertainty bound is always lower than that of the fine-grained entropic uncertainty. And in most cases the Berta’s lower bound would never be achieved, except in some special cases such as Alice and Bob sharing the maximally entangled states. We also get the same conclusion by numerical simulating, varying both *S* and *R* measurements or for different states determined by the parameters *c*_1_, *c*_2_, *c*_3_, and the theoretical results are shown in Supplementary Information. Besides, more numerical simulation results can be obtained from the Mathematica program, as we show in ref.^35^.

Many previous studies have shown that uncertainty principle could be applied in quantum key distibution protocols^6,7,30,36,37^. Using the result of ref.^38^, namely, the lower bound of the amount of key, K, that Alice and Bob are able to extract from the per state *ρ*_*ABE*_ shared by Alice (A), Bob (B) and eavesdropper Eve (E), is *S*(*R*|*E*) − *S*(*R*|*B*), Berta *et al*. derived a new lower bound on the key extraction rate^6^, $K\ge {\mathrm{log}}_{2}\frac{1}{c}-S(R|B)-S(S|B)$. It is worth noting that the minimum of the last two terms of the lower bound, *S*(*R*|*B*) + *S*(*S*|*B*), can be expressed as ${{\rm{\min }}}_{R,S}[H({p}_{d}^{R})+H({p}_{d}^{S})]$ when Alice and Bob chose the same measurement on their respective sides, which enables Pramanik *et al*. to derive a tighter lower bound of the key extraction rate^30^, given by $K\ge {\mathrm{log}}_{2}\frac{1}{c}-[H({p}_{d}^{{\sigma }_{Z}})+H({p}_{inf}^{S})]$. The bound is an optimal lower limit of key extraction, and valid not only for any shared correlation but also for all the measurement setting chosen by Alice and Bob, which may be proved in an experiment of an prototypical quantum key distribution protocol^37,39^.

## Conclusion

In this paper, we have performed an experiment to investigate the fine-grained entropy uncertainty relation using two-qubit state with maximally mixed marginal, and numerically simulated different states by varying state parameters *c*_1_, *c*_2_, *c*_3_. The results show that the fine-graining indeed helps to give a tighter lower bound of the uncertainty relation, which also could be applied in quantum key distribution protocols, deriving an optimized lower bound of the key extraction rate that Alice and Bob are able to obtain, when they perform the same measurements on their respective sides. Besides, our results also may contribute to further knowledge of uncertainty principle.

## Supplementary information

Supplementary information: Experimental test of fine-grained entropic uncertainty relation in the presence of quantum memory
