# Supplementary material for: Experimental test of fine-grained entropic uncertainty relation in the presence of quantum memory
Source: Sci Rep. 2019 Jun 19;9:8748. doi: 10.1038/s41598-019-45205-z (PMC6584628; doi:10.1038/s41598-019-45205-z)
Supplement: Supplementary file 1 — Supplementary information: Experimental test of fine-grained entropic uncertainty relation in the presence of quantum memory [file 41598_2019_45205_MOESM1_ESM.pdf]

Supplementary information: Experimental test of fine-grained entropic uncertainty relation in the presence of quantum memory

Wei-Min Lv, Chao Zhang,* Xiao-Min Hu, Yun-Feng Huang,† Huan Cao,
Jian Wang, Zhi-Bo Hou, Bi-Heng Liu, Chuan-Feng Li,‡ and Guang-Can Guo

The supplementary shows the numerical results of the Berta's uncertainty bound and the fine-grained uncertainty relation. The purple and green curve surfaces indicate the of fine-grained uncertainty and Berta's uncertainty bound respectively. It can be seen from the theoretical results that the Berta's uncertainty bound is always lower than that of the fine-grained entropic uncertainty, and when the system is maxmally entangles state or maximally mixed state, Berta's lower bound would can be achieved.

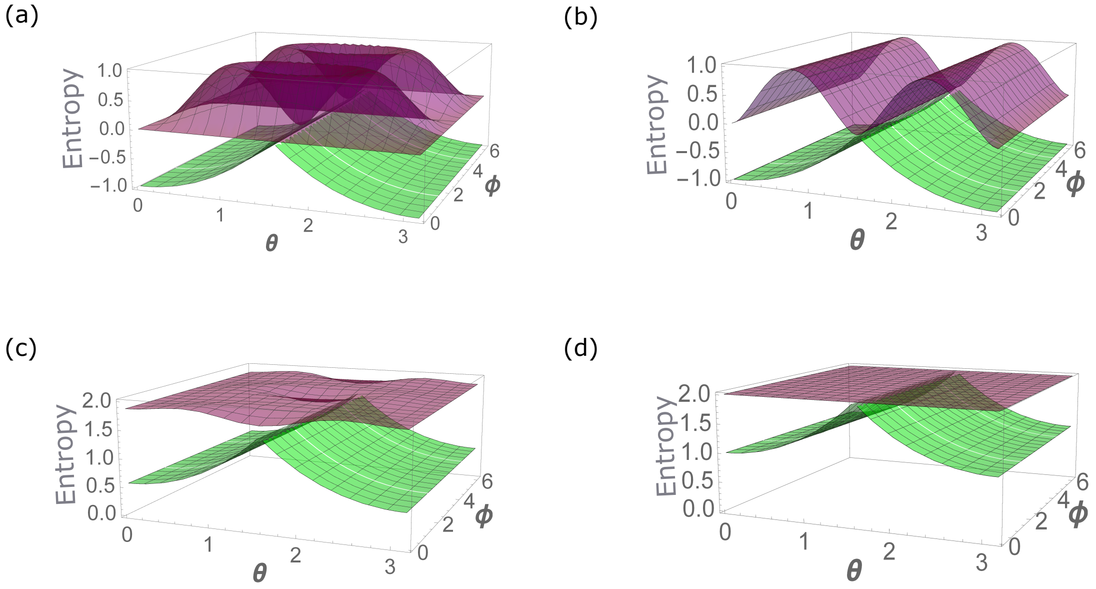

FIG. S1: The theoretical results with different states $\rho = \frac{1}{4} \times \left(I_{4\times4} + \sum_{j=1}^{3} c_j \sigma_j \otimes \sigma_j \right)$ determined by the parameters c_1, c_2, c_3. The purple and green curve surfaces show the theoretical predictions of fine-grained uncertainty $H\left(p_d^{\sigma z}\right) + H\left(p_d^{S}\right)$ and Berta's uncertainty bound $\log_2 \frac{1}{c} + S\left(A|B\right)$ for different θ and ϕ respectively (θ and ϕ are in the unit of radian). (a)$c_1 = 1$, $c_2 = -1$, $c_3 = 1$. ρ=$\frac{1}{\sqrt{2}}\left(|HH\rangle + |VV\rangle\right)$, maximally entangled state. (b) $c_1 = 1$, $c_2 = 1$, $c_3 = -1$, ρ=$\frac{1}{\sqrt{2}}\left(|HV\rangle + |VH\rangle\right)$, maximally entangled state. (c) $c_1 = 0.5$, $c_2 = -0.2$, $c_3 = -0.3$, ρ is a general state. (d) $c_1 = 0$, $c_2 = 0$, $c_3 = 0$, $\rho = \mathbf{I}$, maximally mixed state.

*Electronic address: zhc1989@ustc.edu.cn
†Electronic address: hyf@ustc.edu.cn
‡Electronic address: cfli@ustc.edu.cn

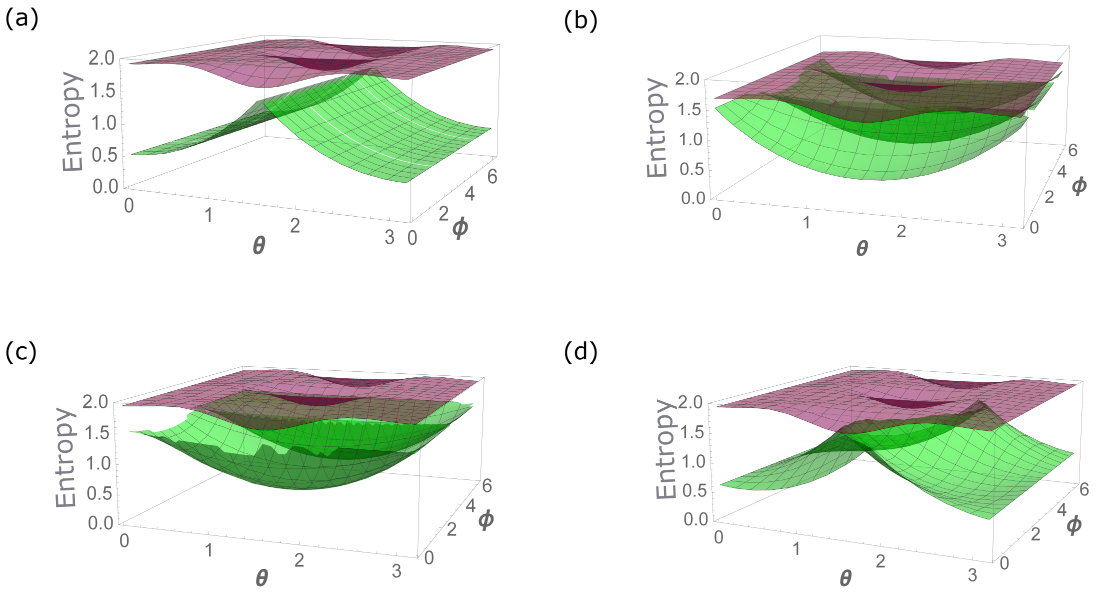

FIG. S2: Theoretical predictions of the the state $\rho = \frac{1}{4} \times \left(I_{4\times4} + \sum_{j=1}^{3} c_j \sigma_j \otimes \sigma_j \right)$ with the parameters $c_1 = 0.6$, $c_2 = -0.16$, $c_3 = -0.24$ for different observable R. (a) $R = \sigma_Z$. (b) $R = \sigma_X$. (c) $R = \sigma_Y$. (d) $R = X(\pi/6, \pi/3)$ where X is determined by the parameters α and β, given that $X = \hat{n} \cdot \vec{\sigma}$, $\hat{n} = \{sin\alpha cos\beta, sin\alpha sin\beta, cos\alpha\}$, $\vec{\sigma} = \{\sigma_x, \sigma_y, \sigma_z\}$ are the Pauli matrices.
